# Twenty Four-Hour Exposure to a 0.12 THz Electromagnetic Field Does Not Affect the Genotoxicity, Morphological Changes, or Expression of Heat Shock Protein in HCE-T Cells

**DOI:** 10.3390/ijerph13080793

**Published:** 2016-08-05

**Authors:** Shin Koyama, Eijiro Narita, Yoko Shimizu, Takeo Shiina, Masao Taki, Naoki Shinohara, Junji Miyakoshi

**Affiliations:** 1 Laboratory of Applied Radio Engineering for Humanosphere, Research Institute for Sustainable Humanosphere, Kyoto University, Uji, Kyoto 611-0011, Japan; narita.eijirou.4e@kyoto-u.ac.jp (E.N.); yoko_shimizu@rish.kyoto-u.ac.jp (Y.S.); shino@rish.kyoto-u.ac.jp (N.S.); miyakoshi@rish.kyoto-u.ac.jp (J.M.); 2 Department of Electrical & Electronic Engineering, Graduate Schools of Science and Engineering, Tokyo Metropolitan University, 1-1, Hachioji, Tokyo 192-0397, Japan; shiina-takeo@ed.tmu.ac.jp (T.S.); masao@tmu.ac.jp (M.T.)

**Keywords:** terahertz (THz), cellular genotoxicity, micronucleus (MN) formation, morphological changes, heat shock protein (Hsp), long-term exposure, human eye cells

## Abstract

To investigate the cellular effects of terahertz (THz) exposure, human corneal epithelial (HCE-T) cells derived from human eye were exposed to 0.12 THz radiation at 5 mW/cm^2^ for 24 h, then the genotoxicity, morphological changes, and heat shock protein (Hsp) expression of the cells were examined. There was no statistically significant increase in the micronucleus (MN) frequency of cells exposed to 0.12 THz radiation compared with sham-exposed controls and incubator controls, whereas the MN frequency of cells treated with bleomycin for 1 h (positive control) did increase significantly. Similarly, there were no significant morphological changes in cells exposed to 0.12 THz radiation compared to sham-exposed controls and incubator controls, and Hsp expression (Hsp27, Hsp70, and Hsp90α) was also not significantly different between the three treatments. These results indicate that exposure to 0.12 THz radiation using the present conditions appears to have no or very little effect on MN formation, morphological changes, and Hsp expression in cells derived from human eye.

## 1. Introduction 

Terahertz (THz) waves span the frequency region from 0.1 to 10 THz and are currently used in many technologies [1,2,3]. Until recently, the THz region was underused due to the limited availability of sources and detectors, but the recent development of various THz sources has enabled the detection of several diseases [4,5,6], as well as scanning for weapons, explosives, and biohazards [7,8,9]. THz technologies have particularly been developed and used for telecommunications [10,11,12]. These new technologies concerning multidisciplinary studies are introduced in a publication by Abbasi et al. [13]. The rapid introduction of THz devices has raised public concern about possible adverse effects on human health [14,15,16]. It was previously reported that exposure to THz radiation increased genomic instability in human lymphocytes [14]. Hintzsche et al. [15] suggested that THz radiation could induce significant spindle disturbance, and Alexandrov et al. [16] presented data showing that the expression of some genes was clearly affected following prolonged broad-band exposure to THz radiation. Although there was a slight increase of the temperature in the study by Alexandrov [16], the effects appear to be non-thermal, and the other two studies [14,15] indicate non-thermal effects of the exposure. On the other hand, Scarfì et al. [17] reported that exposure to THz waves did not induce direct chromosomal damage or alter cell cycle kinetics. Bourne et al. [18] investigated the effects of THz radiation on human keratinocytes and found that it caused no detectable adverse effects on protein levels in cells. Furthermore, it was reported that radiation at 0.380 THz and 2.520 THz did not cause DNA damage and did not affect cellular proliferation in skin cells [19]. Therefore, although there are few studies indicating negative effects of THz exposure, some data do indicate that exposure to THz waves can cause adverse effects. It is therefore necessary to evaluate the influence of low-level and long-term exposure of the human body to THz waves. In this study, we targeted on human corneal epithelial (HCE-T) cells, because THz radiation penetrates into the surface of human skin within about 0.5 mm depth. To investigate the non-thermal effects of THz radiation, we manufactured a device that can expose cells to 0.12 THz radiation. In this study, we used HCE-T cells to examine genotoxicity by micronucleus (MN) frequency, morphological changes using a cell analyzer device, and heat shock protein (Hsp) expression by Western blotting. These metrics are the most studied methods because they are convenient and reliable tests to find the effects of the exposure. 

## 2. Materials and Methods

### 2.1. Exposure Set-Up

**Figure 1:**
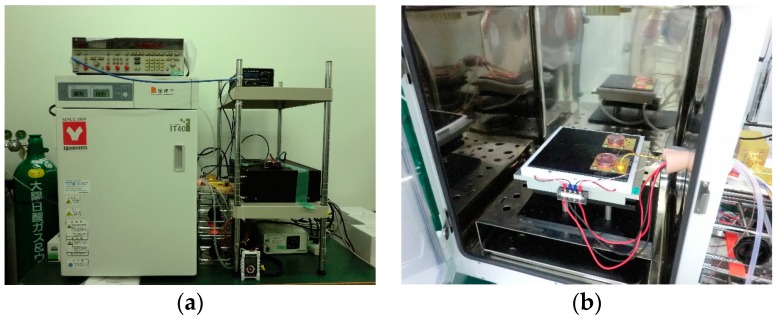
(**a**) The built-in incubator of the 0.12 THz exposure system, and (**b**) an inside view of the incubator. The cavity in the exposure system was maintained under controlled conditions similar to those in an incubator—i.e., an atmosphere of 95% air and 5% CO_2_ at a relative humidity of >95% and a temperature of 37 °C.

We used a specially-designed apparatus that can expose cells to 0.12 THz radiation with a power density of 5 mW/cm^2^ (Figure 1a,b). The details of the exposure system were described previously [20]. Briefly, the applicator comprises a printed circuit board with a large circular exposure area that is fed with THz waves using post-wall waveguide technology. A culture dish is placed on top of the applicator. An electromagnetic field (0.12 THz) is emitted from narrow slots at the top of the applicator and penetrates the culture medium and cells adhering to the bottom of the culture dish. Both the culture dish and applicator were 35 mm in diameter. The electromagnetic field was calculated using commercial finite-difference time-domain (FDTD) software (SEMCAD X version 14.8, Schmid & Partner Engineering AG, Zürich, Switzerland) as the same as at 60 GHz [20]. Simulations were performed using a grid size of 0.05 mm. The relative permittivity and electrical conductivity of the culture medium were 8.75 and 97.7 S/m, respectively, according to Ellison’s model [21] of pure water at 37 °C. The exposures were performed uniformly over the exposure dishes. Most of the cells (76%) are located within the 5 dB region relative to the average specific absorption rate. The penetration depth of the energy in liquid media was 0.3 mm. The chamber of the exposure system was maintained under controlled conditions similar to those in a conventional incubator—i.e., an atmosphere of 95% air and 5% CO_2_ at a relative humidity of >95% and a temperature of 37 °C. The spatially-averaged power density was set to 5 mW/cm^2^ at the bottom of the culture medium where the cells were located. However, the cells were not included in the numerical model. Temperature increases in the dish were controlled to less than 0.3 °C for these conditions.

### 2.2. Cell Culture

The HCE-T human corneal epithelial cell line (RIKEN CELL BANK, Ibaraki, Japan) was kindly supplied by Tokyo Metropolitan University. The cells were maintained in Dulbecco’s Modified Eagle Medium (DMEM) (Wako Pure Chemical Industries, Ltd., Osaka, Japan) plus HamF12 (Wako) (1:1) medium supplemented with 5% fetal bovine serum (FBS) and a final concentration of 5 μg/mL insulin (Sigma-Aldrich, St. Louis, MO, USA) and human epidermal growth factor (Roche, Basel, Switzerland) at a final concentration of 10 ng/mL. The cells were seeded onto 35 mm dishes (Asahi Glass, Tokyo, Japan) at a density of 5 × 10^4^ cells/mL with 2 mL medium. After overnight culture, the cells were exposed to 0.12 THz, and 24 h later the cells were collected and used for experiments. To provide positive controls, the cells were treated with 10 μg/mL bleomycin (Wako) for 1 h (MN frequency), with 2.5 μM latrunculin A (Sigma-Aldrich) for 30 min (morphological changes), or heated at 43 °C for 0.5–2 h (Hsp expression).

### 2.3. MN Formation

The methodology for conducting the MN formation test has been described previously [22]. Briefly, after exposure to 0.12 THz radiation or to 10 μg/mL bleomycin for 1 h, the cells were cultured in medium supplemented with 3 μg/mL cytochalasin B (Sigma-Aldrich) in a conventional incubator for 24 h, centrifuged onto slides using a Cytospin centrifuge (Shandon Southern Instruments Ltd., Cambridge, UK) at 100× *g* for 5 min, fixed with cold 80% ethanol for 30 min, and stained with 0.2 μg/mL propidium iodide (Sigma-Aldrich). A total of 300 binucleated cells were counted, and the frequency of micronucleus formation was determined according to the criteria described previously using a fluorescence microscope (Olympus, Tokyo, Japan). At least six independent tests were performed.

### 2.4. Morphological Changes

Morphological changes were detected using a cell analyzer (xCELLigence System RTCA DP, ACEA Biosciences, Inc., San Diego, CA, USA). The degree of morphological change was determined from the impedance variation (cell index), as previously described [23,24,25]. The cells were exposed to 0.12 THz radiation or latrunculin A (Sigma-Aldrich), then collected using a cell scraper. The cells were re-seeded onto a cell plate coated with fibronectin (Sigma-Aldrich) to measure the cell index caused by the attachment of the cells. After seeding, the cells begin to attach onto the surface of the plate. Following the initiation of correct attachment, the cells extend pseudopods in all directions. However, if attachment is incorrect—for example, due to the inhibition of actin—the cells cannot extend pseudopods. These differences in attachment are complete between 2 and 6 h after seeding. The cell analyzer can detect the attachment of a cell from their impedance. We therefore evaluated the rate of the increase in impedance from 1 to 6 h after the initiation of attachment as the slope, and at least three independent tests were performed.

### 2.5. Hsp Expression

The methodology for quantifying Hsp expression has been described previously [26]. Briefly, after 0.12 THz radiation or heat treatment, the cells were washed with cold PBS, collected using a cell scraper, and the proteins were extracted using CelLytic™ (Sigma-Aldrich) supplemented protease inhibitor cocktail (Sigma-Aldrich). The extracted proteins were incubated at 4 °C for 15 min and centrifuged at 100× *g* for 15 min. The supernatants were collected, and the protein concentrations were measured using an iMark plate reader (Bio-Rad, Hercules, CA, USA) and a calibration curve, and adjusted to 1 mg/mL. The samples were mixed with 2ME (mercaptoethanol) sample buffer (Wako) at a ratio of 1:1 and incubated at 100 °C for 1 min, then immediately put on ice. Extracted protein (20 µg) was loaded onto a 12.5% sodium dodecyl sulfate (SDS)-polyacrylamide gel (Wako), separated by electrophoresis, and transferred to a nitrocellulose membrane (Life Technologies Japan, Tokyo, Japan) using iBlot (Life Technologies Japan). BenchPro™ 4100 (Invitrogen, Carlsbad, CA, USA) was used for blocking and immunostaining. The membrane was blocked with skimmed milk (DS Farma Biomedical, Osaka, Japan) for 1 h and immunostained with antibodies for 1 h. Primary antibody for Hsp27 (R & D Systems, Minneapolis, MN, USA, 1:10,000), Hsp70 (StressMarq Biosciences Inc., Victoria, BC, Canada, 1:1000), Hsp90α (StressMarq, 1:2000), and β-actin (Sigma-Aldrich, 1:1000) were used. The secondary antibodies used were anti-mouse (GE Healthcare, Tokyo, Japan, 1:1000), anti-goat (R & D, 1:500), and anti-rabbit (Sigma-Aldrich, 1:500). After immunostaining, the membranes were stained with horseradish peroxidase, followed by analysis using ATTO Image Analysis Software (Tokyo, Japan). At least three independent experiments were performed.

### 2.6. Statistical Analysis

The data were analyzed using Tukey’s test. *p* values < 0.05 or 0.01 were considered to be statistically significant. The statistical power (1−β) in MN test was calculated using the effect size (f) = 0.1.

## 3. Results

### 3.1. MN Formation

**Figure 2:**
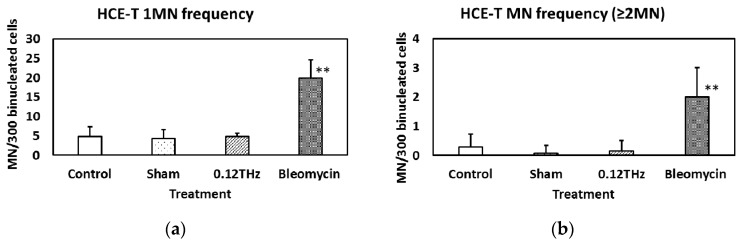
(**a**) Single and (**b**) ≥2 micronucleus (MN) frequency in human corneal epithelial (HCE-T) cells exposed to 0.12 THz radiation for 24 h. The positive control was treatment with bleomycin (10 µg/mL). Data are presented as the mean ± SD from six independent experiments. Asterisks indicate *p* < 0.01. f = 0.1, (1−β) = 0.872.

The frequencies of formation of single MN and two or more (≥2) MN in HCE-T cells are shown in Figure 2a,b, respectively. Both MN frequencies increased significantly upon treatment with bleomycin, whereas no significant difference in either frequency was observed in cells exposed to 0.12 THz radiation, or in the sham-exposed controls and incubator controls. These results suggest that 24-h exposure to 0.12 THz radiation may have no significant effect on MN frequency on HCE-T cells.

### 3.2. Morphological Changes

**Figure 3:**
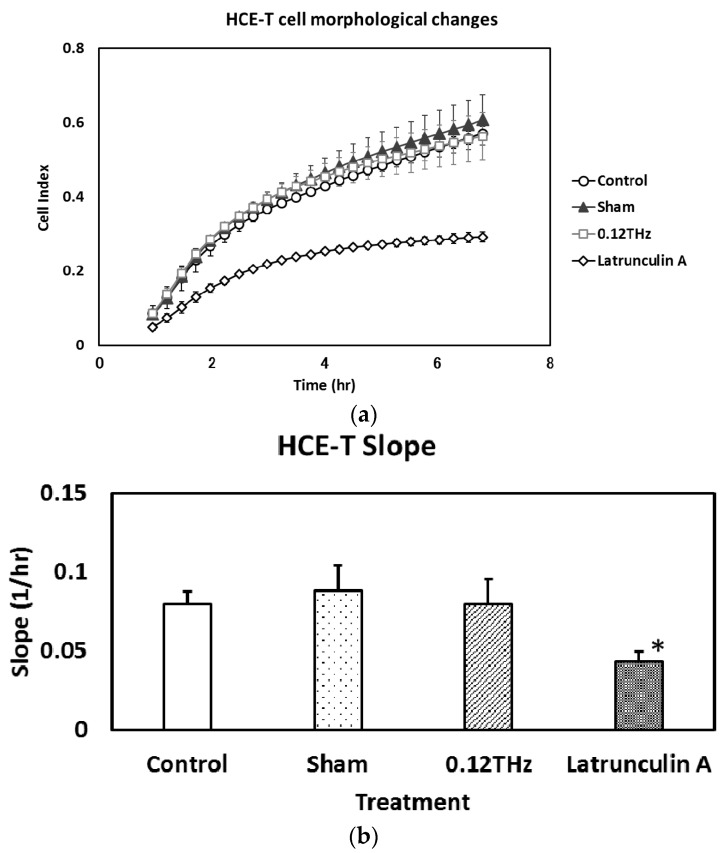
(**a**) The cell index of HCE-T cells and (**b**) the slope of HCE-T cells exposed to 0.12 THz radiation for 24 h. Treatment with latrunculin A (2.5 µM) provided the positive control. Data for the slope are presented as the mean ± SD from three independent experiments. * *p* < 0.05.

The cell index and the slope of the plot of HCE-T cell index vs. time are shown in Figure 3a,b, respectively. The cell index indicates the degree of attachment on the cell plate, and the slope indicates the rate of change in the cell index per hour measured 1 and 6 h after seeding. The cell index and slope decreased significantly following treatment with latrunculin A treatment, whereas there was no significant effect in cells exposed to 0.12 THz radiation or in the sham-exposed controls and incubator controls. These results suggest that 24-h exposure to 0.12 THz radiation may have no significant effect on morphological changes in HCE-T cells.

### 3.3. Hsp Expression

**Figure 4:**
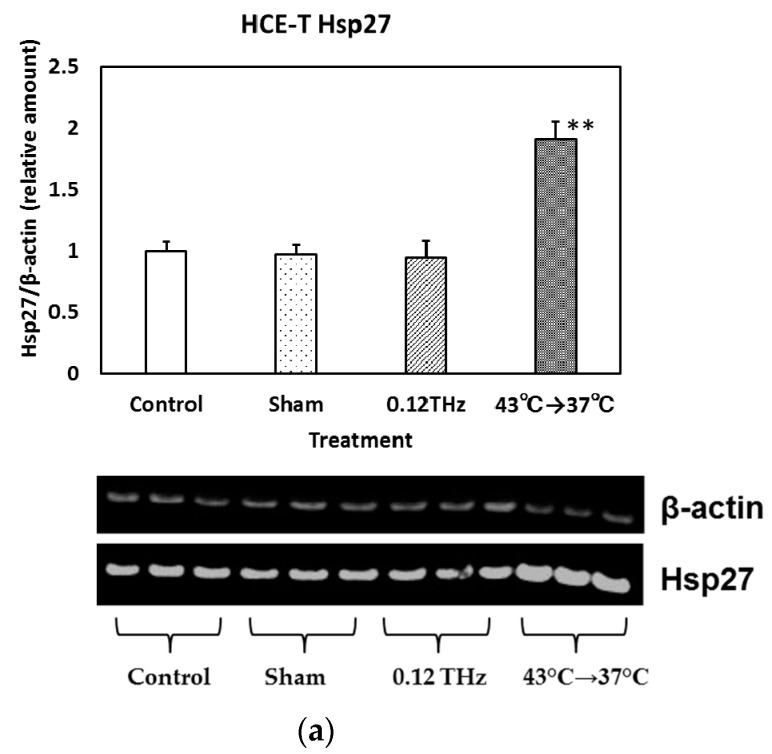

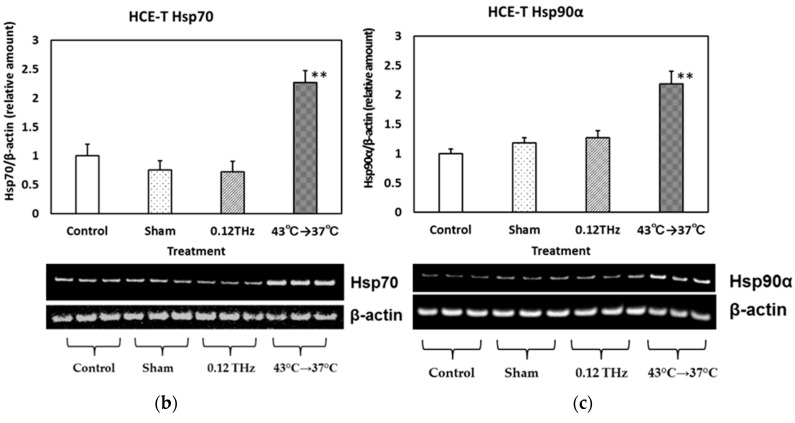
The expression levels of (**a**) heat shock protein 27 (Hsp27); (**b**) Hsp70; and (**c**) Hsp90α in HCE-T cells exposed to 0.12 THz radiation for 24 h. The positive control comprised heat treatment at 43 °C for 0.5–2 h. Data are presented as the mean ± SD from three independent experiments. ** *p* < 0.01.

The expression of Hsp27, Hsp70, and Hsp90α in HCE-T cells is shown in Figure 4a–c, respectively. Heat treatment (Hsp27, Hsp90α: 43 °C (30 min) to 37 °C (6 h), Hsp70: 43 °C (2 h) to 37 °C (1 h)) increased the level of each Hsp significantly. However, no significant difference in any of these proteins was observed in cells exposed to 0.12 THz radiation, or in the sham-exposed controls and incubator controls. These results suggest that 24-h exposure to 0.12 THz radiation may have no significant effect on Hsp27, Hsp70, and Hsp90α expression in HCE-T cells.

## 4. Discussion

A novel apparatus allowing improved and well-defined exposure conditions was used to expose cells to THz radiation. The apparatus provides homogeneous radiation over the exposure area, as well as power efficiency and temperature control of the targeted cells. The temperature increase of the culture medium must be controlled during irradiation to allow analysis of the non-thermal effects of THz exposure, which remain controversial. After confirming the correct exposure conditions, we evaluated the effects of 0.12 THz exposure on MN formation, morphological changes, and Hsp expression in HCE-T cells. Bleomycin-treated cells showed a significant difference in MN formation compared with the incubator control and sham control, whereas 0.12 THz-exposed cells were not significantly different from the controls. The cell index (which indicates morphological changes) of 0.12 THz-exposed cells did not differ from that of the controls, though latrunculin A-treated cells (used as a positive control) showed poor attachment. Expression of Hsp27, Hsp70, and Hsp90α were unchanged among the controls and exposed cells, although heat-treated cells showed increased expression of each Hsp. These data suggest that exposure to 0.12 THz radiation at a power density of 5 mW/cm^2^ does not affect MN formation, morphological changes, or Hsp expression using our experimental conditions.

Terahertz wave applications are increasingly appearing worldwide in various fields, such as diagnosis, security, and telecommunications. It is therefore very important to evaluate the effects of THz exposure at typical usage levels. In our study, we could not detect any adverse effects on cells by exposure to THz waves, in contrast to several earlier studies indicating significant effects of THz exposure. 

The effect of a 94 GHz electromagnetic field on neuronal microtubules was previously investigated [27] and shown to increase the rate of microtubule assembly. However, this increase was attributed by the authors of that paper to be due to an elevation in the temperature of the cell environment caused by the exposure (a rapid electromagnetic field-elicited temperature jump). We evaluated the effects of 0.12 THz (120 GHz) radiation and obtained similar results under controlled temperature conditions. Similarly, Wilmink et al. [28] investigated the biological effects (cellular viability, transcriptional activation of protein and DNA sensing genes) in human dermal fibroblasts exposed to 2.52 THz radiation, and concluded that the observed biological effects could be due to thermal damage, consistent with our findings. 

On the other hand, statistically significant differences were observed for the interaction of protein recognition molecules following THz exposure [29], although the decrease in activity was small. However, it would be a very interesting result if molecular interactions are enhanced by THz exposure. 

Recently, controversial results different from ours were reported [30]. In our study, we could not detect an increase in MN formation following THz exposure, whereas the previous authors indicated that THz exposure (0.1–0.15 THz) induced an increase in the total number of MN and an increase in actin polymerization with no temperature increase of less than 0.2 °C during the exposure. Although their exposure conditions and test cells were different from those used in the current study, the previous authors suggested that their results might support a hypothesis for the abnormal assembly of spindle proteins [19]. 

There are clearly contradictory results concerning THz exposure, and the effects of exposure and temperature must be separated to clarify the mechanism underlying positive non-thermal effects, if any. As we have been performing many investigations at different conditions, we should make better use of mutual exposure devices to adjust the same conditions. In addition, we have to consider rigid statistical calculations which we might be missing. The reliance on *p*-values for the main statistical analysis is a limitation. The difficulty of *p*-values and null hypothesis significance testing have been discussed at length in recent articles in the literature [31].

Fröhlich assumed that THz exposure has a non-thermal effect mediated by the excitation of specific biological macromolecules or linear/nonlinear resonance mechanisms [32]. We are currently conducting other frequency experiments to verify Fröhlich’s hypothesis.

## 5. Conclusions

The data obtained in the present study suggest that exposure to 0.12 THz radiation for 24 h has no significant effect on MN frequency, morphological changes, and the expression levels of Hsp27, Hsp70, and Hsp90α in HCE-T cells. It appears that exposure to 0.12 THz radiation does not cause any adverse effects on MN formation, morphology, or Hsp expression in cultured human eye cells using our experimental conditions.

## Abbreviations

The following abbreviations are used in this manuscript:

THz	Terahertz
HCE-T	Human corneal epithelial
MN	Micronucleus
Hsp	Heat shock protein
